# Impaired Hematologic Status in Relation to Clinical Outcomes among HIV-Infected Adults from Uganda: A Prospective Cohort Study

**DOI:** 10.3390/nu10040475

**Published:** 2018-04-12

**Authors:** Amara E. Ezeamama, David Guwatudde, Alla Sikorskii, Edmond K. Kabagambe, Raybun Spelts, Grace Vahey, Jenifer I. Fenton, Wafaie W. Fawzi

**Affiliations:** 1 Department of Psychiatry, College of Osteopathic Medicine, Michigan State University, East Lansing, MI 48824, USA; Alla.Sikorskii@hc.msu.edu; 2 School of Public Health, Makerere University College of Health Sciences, Kampala, Uganda; dguwatudde@gmail.com; 3 Department of Statistics and Probability, Michigan State University, East Lansing, MI 48824, USA; 4 Division of Epidemiology, Department of Medicine Vanderbilt University Medical Center, Nashville, TN 37203, USA; edmond.kabagambe@vanderbilt.edu; 5 College of Public Health, University of Georgia, Athens, GA 30602, USA; raybun2012@gmail.com (R.S.); grace.vahey@gmail.com (G.V.); 6 Department of Food Science and Human Nutrition, Michigan State University, East Lansing, MI 48824, USA; imigjeni@anr.msu.edu; 7 Departments of Global Health and Population, Nutrition and Epidemiology, Harvard T. H. Chan School of Public Health, Boston, MA 02115, USA; mina@hsph.harvard.edu

**Keywords:** HIV, anemia, ferritin, anemia persistence, ferritin, iron status, clinical outcomes

## Abstract

Impaired hematologic status (IHS) was investigated as a determinant of immune function defined as cluster of differentiation 4 (CD4) T-helper cell count, quality of life (QOL) weight and hospitalization/mortality over 18-months among 398 adult persons living with HIV/AIDS (PLWHA) on anti-retroviral therapy. IHS was defined as having anemia at baseline (Hemoglobin: <12 g/dL for women and <13 g/dL for men), time-updated anemia or having low (<30 μg/L) or high (>200 μg/L for men and >150 μg/L for women) ferritin levels at baseline. Months-to-hospitalization/death or study-end (if no event) was calculated from enrollment. Multivariable linear-mixed models quantified associations between IHS and changes in CD4 cell-count, weight gain and QOL. Cox proportional hazards models calculated hazard ratios (HR) and corresponding 95% confidence intervals (CI) for IHS-related differences in time-to-hospitalization/death. The prevalences of anemia and high and low ferritin levels at baseline were 48.7% (*n* = 194), 40.5% (*n* = 161) and 17% (*n* = 68), respectively. Most patients (63.4%, *n* = 123) remained anemic during follow-up. Weight gained (ferritin-time interaction, *p* < 0.01) and QOL (anemia-time interaction, *p* = 0.05; ferritin-time interaction, *p* = 0.01) were lower for PLWHA with versus without IHS. Relative to anemia-free/normal ferritin, the risk of hospitalization/death was elevated for PLWHA with anemia (HR = 2.0; 95% CI: 1.2–3.6), low or high ferritin (HR: 1.8–1.9, 95% CI: 0.9–4.1) and those that developed new/persistent/progressive anemia (HR: 2.3–6.7, 95% CI: 1.0–12.7). Among PLWHA, IHS predicted deficits in QOL, low weight gain and a high risk of hospitalization/death. Intervention to mitigate persistent IHS may be warranted among PLWHA on long-term highly active antiretroviral therapy (HAART) to improve health outcomes.

## 1. Introduction

Impaired hematologic status (IHS)—including anemia and high or low ferritin—remains highly prevalent in chronic HIV despite highly active antiretroviral therapy (HAART) [1,2,3]. HIV infection leads to IHS through multiple mechanisms including the HIV-1 Nef protein increasing intra-macrophagic iron accumulation [4] and perturbation of acute phase proteins/hormones such as hepcidin, which regulates iron thirst in macrophages [5,6,7]. In return, increased iron leads to increased viral replication through increased reverse transcriptase activity [8], increased availability of iron-dependent enzymes [8] and generation of reactive iron species [9]. The dynamic interactions between HIV and iron create a positive feedback loop that increases the risk of iron overload, onset and persistence of non-iron deficiency anemia and may partly mediate HIV viral persistence and rebound despite HAART [8,10,11]. Persistent immune activation typical in HIV infection contributes to anemia of chronic disease (ACD) [12]. Anemia, regardless of etiology, is associated with sub-optimal immune recovery [13] and mortality [14] in persons living with HIV/AIDS (PLWHA).

Ferritin is commonly measured in epidemiologic studies as biomarker of bio-available iron [11]. High serum ferritin (hyperferritinemia) is commonly described in HIV infection and has been associated with sub-optimal immune recovery and mortality [10,12,15,16]. Low serum ferritin (hypoferritinemia) is seldom described in chronic HIV infection and could occur in the presence of normal red blood cell indices. Hypoferritinemia may result from impaired gastrointestinal nutrient absorption and is associated with folate deficiency, iron deficiency anemia [17] and other morbidity [18] including fatigue and depressed mood [19].

Among PLWHA on long-term HAART, the morbidity significance of IHS–particularly hypoferritinemia and anemia persistence, is poorly understood. This study informs existing knowledge gap by evaluating IHS as a determinant of adverse immunologic and clinical outcomes among adults with chronic HIV-infection stably connected to HIV-care in Kampala, Uganda. It specifically tests the hypothesis that impaired hematologic indices and/or their persistence over time will be associated with worse outcomes in treated HIV despite HAART.

## 2. Materials and Methods

### 2.1. Study Population and Design

This longitudinal study uses data from multiple micronutrient trial (vitamins B, C and E vs. placebo) conducted among HIV-infected Ugandan adults between 2009 and 2012. All participants had World Health Organization (WHO) HIV disease stage 3 or higher, were initiating HAART upon study enrollment or had been on HAART for six months or less. Participants were followed-up every six months for a total of 18 months [20].

### 2.2. Measurements

#### 2.2.1. Primary Determinants: Baseline Serum Ferritin, Baseline Anemia and Anemia Persistence

Ferritin was measured at enrollment only in serum using enzyme-linked immunosorbent assays (ELISA) [21,22]. Per prior precedent, low ferritin was defined as <30 μg/L [21]. High ferritin was defined as >200 μg/L for men and >150 μg/L for women [21]. Anemia was defined at baseline and months 6, 12 and 18 according to WHO guidelines (<12 g/dL for women and <13 g/dL for men) [23]. Anemia severity was graded as mild: 11–11.9 g/dL for women and 11–12.9 g/dL for men; moderate: 8–10.9 g/dL for both sexes; and <8 g/dL as severe anemia for both sexes [23].

#### 2.2.2. Anemia Persistence

Six profiles of time-varying anemia were defined to classify major patterns of anemia evolution within PLWHA and allow explicit comparison of health outcomes among them. Patterns of anemia persistence expected to have a similar degree of impact on clinical outcomes were combined within profiles to enhance statistical efficiency for quantifying the association between this variable and respective outcomes.

(i) Persistently anemia free: no anemia in any observation period.
(ii) Completely Resolved Anemia: mild or moderate/severe anemia at enrollment only without evidence of anemia in any follow-up interval.
(iii) Incompletely Resolved Anemia: baseline moderate/severe anemia that was down-modulated to mild in ≥1 follow-up interval.
(iv) Incident moderate/severe or sustained mild anemia: baseline anemia that became moderate/severe in one of three follow-up intervals or mild baseline anemia that was sustained in ≥2 follow-up intervals.
(v) Mild Anemia: one episode of mild anemia at baseline or developed mild anemia in one of the follow-up intervals.
(vi) Progressive or Persistent moderate/severe Anemia: baseline moderate/severe anemia that was sustained in ≥2 assessment intervals or mild baseline anemia that progressed to moderate/severe in ≥1 follow-up intervals.

#### 2.2.3. Outcomes

Four outcome variables were each assessed at months 0, 6, 12 and 18.

(i) Immune function: cluster of differentiation 4 (CD4) T-helper cell count: absolute T-cell lymphocyte count in cells/micro liter was measured using a FACS Calibur flow cytometer (Becton-Dickinson, San Jose, CA, USA).
(ii) Body mass index (BMI): was calculated as the ratio of weight (in kilograms) to squared height (in meters).
(iii) QOL: assessed with the Medical Outcomes Study HIV Health Survey translated and culturally adapted for the study area [24]. As described previously [25], overall QOL score was the sum of scores for activities of daily living, cognitive function, emotional and physical health subscales. Overall score was linearly transformed so that the highest score would be 100.
(iv) Death/hospitalization: Occurrence of hospitalization or death event was, for each participant, a composite endpoint to enhance statistical efficiency and provide a more complete picture of serious adverse clinical outcomes among PLWHA, which should become rarer in the HAART era. Censoring was at date of first hospitalization or death. Participants that experienced both events were censored at hospitalization.

### 2.3. Potential Confounders: Clinical, Socio-Demographic and Behavioral Characteristics

Age was measured in whole years categorized by the approximate quartiles of age distribution in our sample as: 18–29, 30–35, 36–41 and 42+. Socioeconomic status was defined by years of education, employment status and household wealth or assets owned. Composite scores were categorized at quintiles [26]. History and current use of alcohol and cigarette smoking were self-reported.

### 2.4. Statistical Analysis

In addition to baseline assessment, three repeated measures of CD4 + T-cell count, BMI and QOL (6, 12 and 18 months) were analyzed using multivariable linear mixed effects models in relation to baseline ferritin status and baseline anemia (including anemia severity). An unstructured covariance matrix was assumed to accommodate non-independence of repeated outcome assessments within participants. A random intercept was specified to allow within-subject differences in outcome trajectory from enrollment. Empirical standard errors were used to derive robust measures of variance and to mitigate potential misspecification of the covariance matrix. We tested the null hypothesis of no hematologic status related differences in absolute CD4, BMI and QOL over 18 months at *α* = 0.05. In addition, we estimated differences in outcomes according to high or low versus normal baseline ferritin, baseline anemia and baseline anemia-severity versus no anemia at months 0, 6, 12 and 18. Next, anemia persistence variable was used as an explanatory variable in the regression analysis of each outcome (absolute CD4, BMI and QOL) at month 18. We constrained this analysis only to month 18 to ensure temporal sequence between evolution of anemia severity since enrollment and respective outcome measures. Lastly, we used Cox regression to test whether baseline ferritin level, baseline anemia (including severity) and anemia persistence were associated with time to hospitalization or death over 18 months of follow-up.

To address possible confounding by extraneous covariates, we adjusted the analyses for baseline measures of behavioral factors (smoking and drinking), socio-demographic characteristics (age at enrollment, sex, income and marital status), parent study trial arm, baseline HAART experience and vitamin D status. Final multivariable regression models included independent predictors of respective outcomes and candidate confounders as described in the literature. We adjusted for baseline CD4 and BMI in models where they are not primary outcomes. All analyses were implemented in Statistical Analyses Software (SAS) version 9.4 (SAS Institute, Cary, NC, USA).

### 2.5. Consent Process/Ethical Approval

At enrollment in the parent study, each participant provided written informed consent administered in the local language of Luganda. The study was approved by the Scientific Review Committee of the Infectious Diseases Institute at Makerere University College of Health Sciences and the Institutional Review Boards of Harvard School of Public Health (protocol number: 17361) and that of Makerere University School of Public Health (protocol number: HDREC 067). The study was further registered and approved by the Uganda National Council for Science and Technology (UNCST, protocol number: HS 629).

## 3. Results

### 3.1. Baseline Characteristics

Low, normal and high ferritin groups were similar in terms of material wealth, prevalence of alcohol use, multivitamin use, mean CD4 cell count, vitamin D level, employment/work status, QOL score and education. Participants with high ferritin group tended to be older, have lower BMI, higher C-reactive protein (CRP) and slightly more anemia. Participants with low ferritin tended to be younger, include more females and were less likely to rate current health as good, very good or excellent. (Table 1).

BMI, hemoglobin values and QOL scores increased steadily over the entire study period. Conversely, anemia prevalence (and the severity of anemia) decreased from 48.5% at enrollment to 22.7% by month 18 (Table S1). By study end, nearly 30% (*n* = 58) of baseline anemia was completely resolved and another 17.5% (*n* = 34) was only partially resolved. However, anemia developed after enrolment in 14.7% (*n* = 30) of PLWHA without baseline anemia. Anemia persisted at some level for most PLWHA with baseline anemia (*n* = 119, 63.4%), including 19.6% (*n* = 38) for those whose anemia either progressed from mild to moderate/severe or was sustained at moderate/severe from enrollment (Table S2).

### 3.2. Association between Baseline Ferritin Status and Immune Recovery, BMI and QOL Outcomes

Baseline ferritin showed no association with absolute CD4 cell count over the study period (ferritin × time, *p* = 0.78) (Table 2). However, change over time in BMI and QOL varied by baseline ferritin status (ferritin-by-time interaction, *p*-values of 0.04 and 0.01, respectively). Specifically, patients with low or high ferritin status had no significant difference in weight gain compared to patients of normal ferritin status during months 0 and 6. By month 12 and 18 however, patients with low or high baseline ferritin gained between 0.2 and 0.5 kg/m^2^ more weight than those with normal baseline ferritin. This superior weight gain was only significant for high versus normal ferritin in the interval between 12 and 18 months. QOL scores over time was lower for persons with high or low versus normal baseline ferritin at three out of four follow-up intervals. Low versus normal baseline ferritin related deficits in QOL scores (difference = −3.3, 95% CI: −6.1, −0.6) was statistically significant at month 18 only.

### 3.3. Baseline Anemia/Anemia Severity and Change in Immune Recovery, Weight Gain, QOL

Over 18-month of follow-up, the relationships between CD4 counts did not significantly vary by anemia (data not shown, *p*-value for anemia × time = 0.56) or its severity (*p*-value for anemia severity × time interaction = 0.11) at baseline. The change in weight gain and QOL over time differed by baseline anemia severity. Specifically, at enrolment, PLWHA with severe anemia (difference = −1.3 kg/m^2^, 95% CI: −2.4, −0.3) weighed significantly less than anemia-free PLWHA. The magnitude of baseline weight difference was lower and statistically insignificant for those with mild versus no anemia. During follow-up, all participants gained weight and magnitude of baseline anemia status dependent differences in weight gain declined and became statistically insignificant from study month six. The trend in QOL change by baseline anemia groups was similar to that for weight gain. Specifically, the evidence for anemia dependent differences in QOL existed at baseline only for persons with moderate/severe versus no anemia (QOL difference = −4.5, 95% CI: −4.4, −0.9). Post-enrolment, anemia-dependent deficit in QOL was either reversed in direction or its magnitude was substantially smaller relative to baseline (Table 2).

### 3.4. Association between Anemia Persistence, Immunologic Status and QOL

Anemia persistence was significantly and adversely associated with magnitude of CD4 cell count recovered (*p* < 0.01) and the amount of weight gained (*p* < 0.01) at month 18 (Table 3). PLWHA with sustained moderate/severe or progressive anemia had a substantial deficit in CD4 cell counts compared to PLWHA without anemia (difference = −73 cells/uL, 95% CI: −126 to −20 cells/uL). Similarly, PLWHA with sustained mild anemia in multiple follow-up intervals or who developed an episode of moderate/severe anemia since enrollment had significantly lower month 18 CD4 cell count compared to PLWHA without anemia (difference = −46.2, 95% CI: −91.2 to −1.3). Though statistically insignificant, complete resolution of baseline anemia was associated with gains in absolute CD4-cell count (difference = 27.4, 95% CI: −16.8, 71.5) whereas incomplete anemia resolution was associated with lower month 18 CD4 cell-count (difference = −34.6, 95% CI: −80, 20.9) relative to anemia free PLWHA. The resolution of anemia was positively associated with amount of weight gained by month 18. Specifically, compared to PLWHA without anemia in any interval, those whose anemia totally resolved gained 1.1 kg/m^2^ (95% CI: 0.5, 1.8) and those whose anemia partially resolved gained 1.4 kg/m^2^ (95% CI: 0.6, 2.6) more weight. There was no association between anemia persistence and QOL at month 18.

### 3.5. Time to Hospitalization/Death

The incidence rates for death or hospitalization were 6.9 events/1000 person-months in the normal ferritin group, 10.3 events/1000 person-months in the low ferritin group and 12.9 events/1000 person-months in the high ferritin group. For PLWHA with and without baseline anemia, the incidence of death/hospitalization were 13.8/1000 person months and 6.2/1000 person-months respectively. The incidence of hospitalization/death increased dramatically from 5.4/1000 person-months among anemia-free participants to as high as 26/1000 person months for participants with persistent moderate/severe or progressive anemia (Table 4).

**Figure 1:**
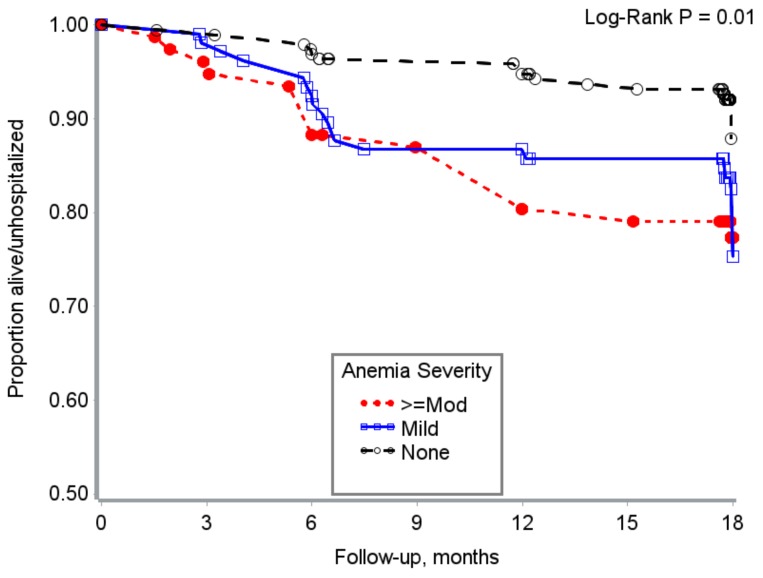
Hospitalization free survival time by baseline anemia severity among HIV + adults from Uganda.

**Figure 2:**
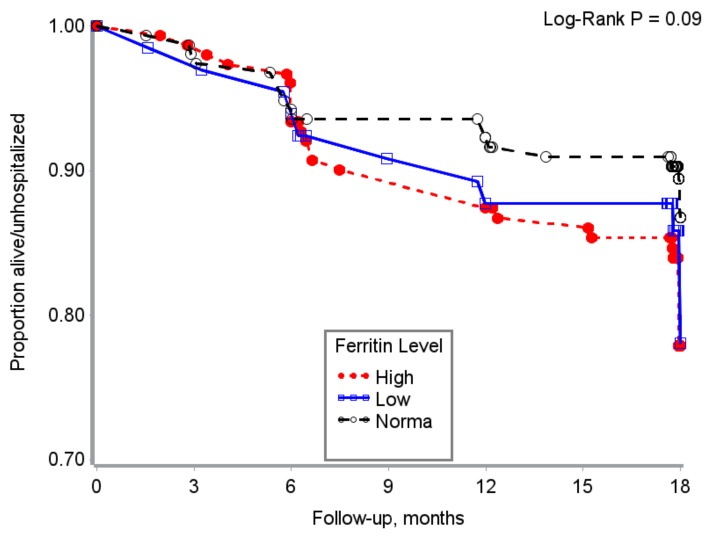
Serum ferritin status and hospitalization free survival time in HIV + adults from Uganda.

Hospitalization free survival time varied by baseline anemia severity and measures of anemia persistence (Figure 1 and Figure 2).

**Figure 3:**
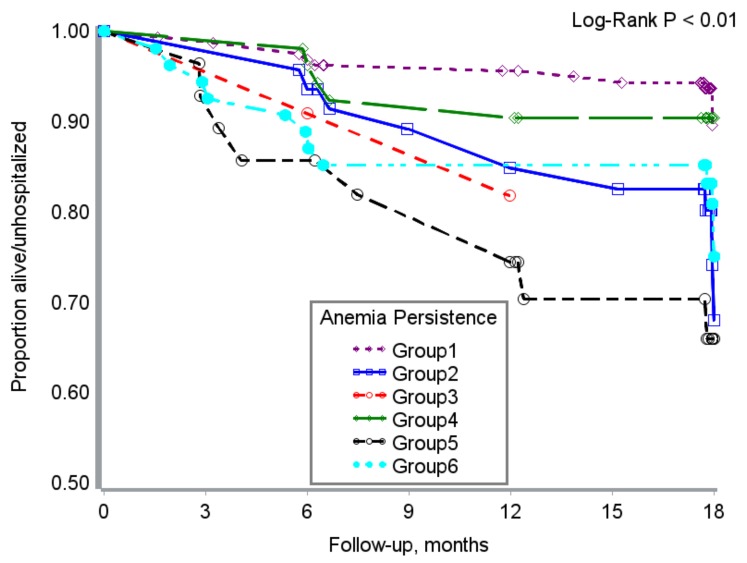
Severity of anemia persistence from enrollment and hospitalization-free survival time in HIV + adults from Uganda. Group 1 = adults anemia free in all intervals; Group 2 = Participants with baseline mild or moderate/severe anemia completely resolved during FU. Group 3 = Baseline moderate/severe anemia incompletely resolved (i.e., down-modulated to mild anemia); Group 4 = Developed single episode of moderate anemia or had baseline mild anemia that persisted in two or all three follow-up intervals. Group 5 = Baseline mild anemia only or developed single episode of mild anemia in one of three follow-up intervals. Group 6 = participants with baseline moderate/severe anemia that persisted during follow-up or participants with mild baseline anemia progressed to moderate/severe during one or more follow-up intervals.

Hospitalization free survival time was marginally lower for PLWHA with high or low versus normal ferritin (Figure 3).

In Cox proportional hazard models, high or low ferritin levels were associated with 75–85% higher rates of death over the study period but these associations were statistically insignificant after adjustment for confounders. Compared to patients without anemia, individuals with anemia were two times more likely die or be hospitalized. The severity of baseline anemia further augmented risk of hospitalization/death in that those with moderate/severe anemia at baseline were 6.7 times more likely to die or be hospitalized (95% CI: 3.6–12.7) than those with no anemia.

## 4. Discussion

Among adult PLWHA from Uganda, IHS was highly prevalent at or near HAART initiation with 48.7 anemic, 17% with hypoferritinemia and 40.5% with hyperferritinemia at enrolment in this study. Anemia prevalence rate is slightly lower than the 69% and 70.5% rate respectively reported among adult HIV+ Congolese and South Africans at HAART initiation [1,27]. On the other hand, prevalence hyperferritinemia noted is nearly four times as high as noted in HIV+ adults from Spain [10], comparable to 48% previously reported among Tanzanian patients some of whom were HIV and tuberculosis co-infected [28] but lower than the 67% reported among HIV-infected Indonesian adults where prevalence of injection drug use and hepatitis C coinfection were high. The prevalence of hypoferritinemia in this study is in-line with the 16% prevalence noted in Thai patients [29] but somewhat lower than the 6.3–9% observed among Tanzanian and Indonesian adults with HIV [12,28]. Regardless of study setting, evidence from this and prior investigations demonstrate a high burden of IHS among PLWHA. The variations in anemia and ferritin levels by region partly reflect the expected variations based on the characteristics of the study sample, the HAART regimen, HAART duration and the effectiveness of virologic control given HAART.

In line with our study hypothesis, participants with baseline anemia had relative disadvantage with respect to weight gain and QOL at enrolment. Over the study period, as participants stabilized on HAART, these baseline anemia-related deficits in weight gain and QOL outcomes were strongly down-modulated. The contributory role of HAART for resolving pre-HAART anemia in PLWHA has been described [2,3]. Indeed, we document complete or partial resolution of anemia in 47.4% of PLWHA with anemia at enrolment. The resolution of anemia and its salutary impact on wellbeing is corroborated by results from a clinical trial of epoeitin alfa for anemia in US adults [30] and two longitudinal studies of HIV-infected South African [1] and Congolese adults [27]. Anemia resolution in this study likely contributed to the observed progressive decline in anemia related deficits with respect to weight gain and QOL over the study period [1,2].

Our data further suggests that PLWHA who have extreme ferritin levels experience more weight gain by study month 12 than those having normal ferritin levels. This association was statistically robust only among persons with baseline hyperferritinemia, an observation that we speculate is explained by relative changes in the etiology of anemia among persons on long-term HAART. This is the subject of ongoing investigation in our group. Briefly, our unpublished data shows that burden of ACD is greatest in this group (47%) at enrollment compared to participants with low (19.5%) and normal ferritin levels (40.2%). Equally relevant for explaining the differential weight gain among participants with high baseline ferritin, our unpublished data show that the resolution of ACD is highest among baseline hyper-ferritinemic participants by study month 18. Hence, HAART driven resolution of ACD will enable this group experience superior weight gains in the long term. We also noted that baseline low versus normal ferritin was associated with lower QOL by study end. This observation is consistent with the predominance of iron deficiency anemia, folate deficiency and other anemia of nutritional origin in this group. These kinds of anemias maybe beneficially impacted by iron and micronutrient supplementation but not necessarily HAART. Prior work has demonstrated that release of dietary iron from enterocytes is inhibited in the context of inflammation that is characteristic of HIV disease [6,7]. Hence, as gross inflammation is down-modulated on antiretroviral therapy, the importance of anemia of nutritional origin may rise. Furthermore, our data suggests that high or low ferritin level is associated with similarly elevated risk of hospitalization/mortality. These associations are likely clinically relevant given consistency with prior literature–particularly for high ferritin status [17].

Relationships between ferritin categories and most outcomes investigated demonstrated a U-shaped response where associations with morbidity/mortality were similar in direction for PLWHA with high or low compared to normal ferritin. Low ferritin and anemia is expected to increase fatigue, irregular heartbeat and depressed mood [19], whereas high ferritin is expected to enhance the risk of iron overload, metabolic syndrome, insulin resistance and death in PLWHA [31]. Empirical data on the clinical significance of hypoferritinemia in HIV is particularly limited and yet it is quite prevalent for example, 17% in the current study. Findings from this study are consistent with observations among Tanzanian PLWHA where hyperferritinemia was associated with mortality and low-ferritin predicted elevated risk of TB-treatment failure and TB-recurrence [28]. This study highlights the dual burden of iron deficiency anemia and ACD in PLWHA on long-term HAART. Whether iron supplementation in this population will be safe, efficacious and effective is an empirical question at this time. Specific future investigations with repeated measures of hemoglobin and iron-status are needed in order to understand the dynamic change in hematologic status among PLWHA on HAART, morbidities associated with persistent IHS and the possible health and QOL benefits of iron supplementation in hypoferritinemic PLWHA.

Time-varying anemia severity–particularly the development of moderate anemia, progressive, persistent and incomplete resolution of existing anemia, consistently predicted lower immune recovery by study end. Conversely, resolution of existing anemia–even when incomplete, was associated with substantial weight gain advantage in comparison with persistently anemia free patients. Our finding that non-hospitalized survival time was significantly higher and the risk of hospitalization or death was significantly elevated for persons with poor hematologic profile is internally consistent with associations noted herein with soft morbidity indicators–weight gain, CD4 cell count and QOL and with extant literature [3]. Few prior investigations among PLWHA on HAART have focused on resolution of anemia as a key variable [1,27,32] and the relevance of this phenomenon for clinical outcomes to the best of our knowledge has not explored [1,27,32]. We confirm these previously reported findings of HAART mediated anemia resolution and extend current knowledge base by demonstrating the clinical relevance of persistent/unresolved anemia in chronic HIV.

Strengths of this investigation include prospective design that permits temporal inference, a long follow-up duration with repeated assessment of hemoglobin, limited loss of participants to follow-up and control for potential confounders. The emphasis on hospitalization events and typical morbidities—weight loss, QOL and immune recovery appropriate for the post-HAART era of chronic HIV are additional strengths. However, our data does not allow for specific understanding of the predominant forms of anemia that develop or persist in HIV-positive persons on HAART. For example, is it possible that with extended time on treatment and expected down-modulation of severe inflammation that emergent anemia and/or anemia that persists in treated HIV is increasingly iron deficiency anemia and nutritional in etiology? Future prospective studies to understand variations in causes of anemia over time on HAART among PLWHA will crucially inform the appropriate clinical course of action for managing anemia in this population.

## 5. Conclusions

In summary, we confirm previously reported observations of a high IHS burden among PLWHA on HAART and the spontaneous resolution of anemia over 18 months on HAART for some PLWHA. We extend current knowledge base by providing novel empirical data on the adverse impact of persistent anemia on immune recovery, hospitalization and mortality. Of equal importance, we show that resolution of baseline anemia was associated with comparable immune recovery, QOL and risk of hospitalization/death over 18 months relative to PLWHA anemia free throughout. We conclude that intentional management of anemia of all etiologies in chronic HIV-infection presents a key opportunity to enhance functional survival in the increasing number of PLWHA on long-term HAART. Our data shows that despite spontaneous resolution of anemia for some, anemia remains highly prevalent in PLWHA over 18 months on HAART. With antiretroviral therapy-related down-modulation of high level inflammation, the etiology of persistent anemia differs relative to pre- HAART. This possibility should be specifically investigated in future studies among PLWHA on HAART. Given the high prevalence of persistent anemia and the possibility that these could be nutritional or iron-deficiency in etiology, future iron supplementation interventions to clarify the safety, efficacy and proper targeting of iron supplements in PLWHA may be warranted. Such trials should ideally lag HAART initiation and begin when the level of inflammation in PLWHA has been down-modulated by HAART to maximize benefit derived from iron supplementation and minimize the risk of iron-overload.

## Figures and Tables

**Table 1:** Baseline socio-demographic and clinical characteristics of study sample.

Characteristics	Overall	Low Ferritin *n* = 68	Normal Ferritin *n* = 169	High Ferritin *n* = 161	*p*-Value
	*n* (%)	*n* (%)	*n* (%)	*n* (%)	
Age (years)					<0.01
18–29	98 (24.6)	26 (38.2%)	50 (29.6%)	22 (13.7%)	
30–35	104 (26.1)	20 (29.4%)	41 (24.3%)	43 (26.7%)	
36–41	102 (25.6)	15 (22.1%)	39 (23.1%)	48 (29.8%)	
42+	94 (23.6)	7 (10.3%)	39 (23.1%)	48 (29.8%)	
Female	275 (69.1)	68 (100.0%)	134 (79.3%)	73 (45.3%)	<0.01
Clinical Measures					
High C-reactive protein (CRP)	55 (13.9)	4 (5.97%)	15 (8.88%)	36 (22.50%)	<0.01
HAART naïve	199 (50)	36 (52.94%)	95 (56.21%)	68 (42.24%)	0.03
Anemia Severity					0.03
None	204 (51.3)	44 (64.7)	88 (52.1)	72 (44.7)	
Mild	113 (28.4)	10 (14.7)	48 (28.4)	55 (34.2)	
Moderate/Severe	81 (20.3)	14 (20.6)	33(19.5)	34 (21.1)	
Behavioral Factors					
Ever smoked cigarette	69 (17.3)	0 (0.0%)	26 (15.4%)	43 (26.7%)	<0.01
Current Alcohol use					0.61
Never Used	84 (21.1)	16 (23.5%)	40 (23.7%)	28 (17.4%)	
Former User	230 (57.8)	37 (54.4%)	97 (57.4%)	96 (59.6%)	
Current User	84 (21.1)	15 (22.1%)	32 (18.9%)	37 (23.0%)	
Baseline Multivitamin Use	90 (22.6)	17 (25.0%)	34 (20.1%)	39 (24.2%)	0.59
BMI (kg/m^2^)					0.07
Underweight (BMI < 18.5)	22 (5.5)	2 (2.9%)	8 (4.7%)	12 (7.5%)	
Normal (18.5 ≤ BMI < 25)	262 (65.8)	42 (61.8%)	103 (61.0%)	117 (72.7%)	
Overweight (25 ≤ BMI < 30)	72 (18.1)	15 (22.1%)	36 (21.3%)	21 (13.0%)	
Obese (BMI ≥ 30)	42 (10.6)	9 (13.2%)	22 (13.0%)	11 (6.8%)	
Vitamin D Deficiency					0.57
Deficient	67 (16.9)	12 (17.9%)	25 (14.8%)	30 (18.8%)	
Insufficient	239 (60.4)	42 (62.7%)	99 (58.6%)	98 (61.3%)	
Sufficient	90 (22.7)	13 (19.4%)	45 (26.6%)	32 (20.0%)	
Self-rated Health					0.03
Good, Very good or Excellent	180 (45.2)	21 (30.9%)	83 (49.1%)	76 (47.2%)	
Education					0.88
<Primary	165 (41.6)	26 (38.2%)	73 (18.4%)	66 (41.0%)	
Primary completed	54 (13.6)	13 (19.1%)	21 (12.5%)	20 (12.4%)	
Some O’level	78 (19.7)	12 (17.7%)	32 (19.1%)	34 (21.1%)	
O’level or higher	100 (25.2)	17 (25.0%)	42 (25.0%)	41 (25.5%)	
Unemployed/No Income	52 (13.1)	11 (16.18%)	21 (12.43%)	20 (12.4%)	0.77
Continuous Measures	Mean (standard deviation (SD))	Mean (SD)	Mean (SD)	Mean (SD)	
Age (in years)	35.8 (9.0)	31.5 (7.1)	35.1 (8.4)	38.1 (9.5)	<0.01
BMI (in kg/m^2^)	23.8 (4.4)	24.5 (4.2)	24.4 (4.8)	22.8 (3.8)	<0.01
QOL Score	91.2 (9.9)	90.4 (10.2)	91.3 (9.9)	91.5 (10)	0.77
CD4 cell count (cells/µL)	149 (96)	161 (103)	152.1 (94)	140.7 (95)	0.25
Hemoglobin (mg/dL)	12.3 (1.9)	12.3 (1.76)	12.3 (1.8)	12.2 (1.9)	0.68
Vitamin D (ng/mL)	26.7 (7.0)	26.0 (7.5)	26.4 (6.8)	27.3 (7.1)	0.37

**Table 2:** Adjusted mean absolute CD4, body mass index and quality of life score at each study time point in relationship with baseline ferritin & anemia severity.

Outcome Hematologic Status Indicator		Month 0 Mean ± SE Diff (95% CI)	Month 6 Mean ± SE Diff (95% CI)	Month 12 Mean ± SE Diff (95% CI)	Month 18 Mean ± SE Diff (95% CI)	*p*-Value Group × Time
Absolute CD4	Baseline Serum Ferritin					
	Normal ^†^	152.7 ± 6.8	231 ± 8.0	251 ± 9.4	287 ± 10.9	0.78
	High	−7.3 (−29.0, 14.4)	0.3 (−16.4, 17.0)	15.3 (−4.9, 35.4)	16.3 (−12.8, 45.5)	
	Low	−7.0 (−34.7, 20.6)	−6.6 (−30.1, 16.9)	8.1 (−19.1, 35.2)	−4.0 (−37.4, 29.5)	
	Baseline Anemia Severity					
	No Anemia	148 ± 6.8	227 ± 8.0	261 ± 9.4	293 ± 10.9	0.11
	Mild	15 (−8.6, 38.4)	20 (−8, 39)	−1 (−31, 30)	−8 (−44, 27)	
	Moderate/Severe	−14 (−36.4, 9.0)	−9 (−37, 9)	−7 (−41, 28)	9 (−34, 52)	
BMI	Baseline Serum Ferritin					
	Normal ^†^	23.7 ± 0.3	24.3 ± 0.2	24.3 ± 0.2	24.8 ± 0.2	0.04
	High vs. Normal	0.0 (−0.4, 0.4)	−0.1 (−0.4, 0.2)	0.5 (0.2, 0.8)	0.3 (−0.1, 0.7)	
	Low vs. Normal	−0.1 (−0.6, 0.4)	−0.1(−0.4, 0.3)	0.2 (−0.2, 0.6)	0.4 (−0.2, 0.9)	
	Baseline Anemia Severity					
	No Anemia	24.3 ± 0.3	24.5 ± 0.3	24.± 0.3	25.0 ± 0.3	
	Mild	− 0.9 (−1.9, 0.1)	−0.5 (−1.5, 0.5)	−0.3 (−1.3, 0.7)	−0.3 (−1.3, 0.8)	<0.01
	Moderate/Severe	−1.3 (−2.4, −0.3)	−0.8 (−1.8, 0.2)	−0.4 (−1.4, 0.6)	0.1 (−1.0, 1.2)	
QOL	Baseline Serum Ferritin					
	Normal ^†^	91.4 ± 0.7	98.0 ± 0.6	97.6 ± 0.6	98.9 ± 0.6	0.01
	High vs. normal	−0.2 (−2.2, 1.8)	−1.6 (−3.6, 0.1)	1.0 (−0.60, 2.64)	−0.5 (−2.1, 1.2)	
	Low vs. normal	−0.3 (−2.96, 2.28)	−0.1 (−2.1, 1.9)	1.6 (−0.46, 3.50)	−3.3 (−6.1, −0.6)	
	Baseline Anemia Severity					
	No Anemia	91.4 ± 0.7	98.0 ± 0.6	97.6 ± 0.6	98.9 ± 0.6	<0.01
	Mild	−1.1 (−3.3, 1.2)	−0.6 (−2.8, 1.6)	0.9 (−0.8, 2.6)	−1.9 (−4.2, 0.4)	
	Moderate/Severe	−4.5 (−3.5, −0.9)	1.0 (−1.3, 3.3)	−0.9 (−3.9, 2.1)	1.3 (−0.9, 3.5)	

Results are from linear mixed effects models for repeated measures of CD4, BMI and QOL as outcomes. Adjusted least square means and standard error (SE) for the reference (^†^) hematologic status category are shown. For non-reference exposure categories, mean difference from reference category and associated 95% confidence intervals are presented. Analyses are adjusted for age, sex, wealth, CRP, HAART experience at enrollment, multivitamin use, alcohol use & smoking. Baseline CD4 cell count and baseline BMI are further adjusted in models in which these are not dependent variables. Anemia and ferritin levels are not mutually adjusted for one another in any model.

**Table 3:** Persistence of anemia in relationship to clinical and immunologic indicators at month 18 follow-up.

Change in Anemia from Enrolment	Absolute CD4 Cell Count	Body Mass Index *	Quality of Life
Anemia Persistence	Adjusted Mean ± SE	Adjusted Mean ± SE	Adjusted Mean ± SE
	Mean Difference (95% CI)	Mean Difference (95% CI)	Mean Difference (95% CI)
Group 1 (*n* = 166) ^†^	309 ± 11.6	24.7 ± 0.17	98.0 ± 0.67
Group 2 (*n* = 58)	27.4 (−16.8, 71.5)	1.14 (0.49, 1.80)	−0.26 (−2.8, 2.3)
Group 3 (*n* = 35)	−34.6 (−80.0, 20.9)	1.42 (0.61, 0.60)	2.4 (−0.8, 5.7)
Group 4 (*n* = 55)	−46.2 (−91.2, −1.26)	−0.37 (−1.02, 0.28)	−0.47 (−0.31, 2.1)
Group 5 (*n* = 19)	−48.9 (−118.6, 20.9)	−0.40 (−1.40, 0.60)	2.1 (−1.93, 6.2)
Group 6 (*n* = 39)	−72.8 (−125.7, −19.8)	0.23 (−0.54, 1.01)	−2.1 (−5.2, 1.04)
*p*-value (Log Rank test)	0.01	<0.0001	0.24

Group 1 = adults anemia free in all intervals; Group 2 = Participants with baseline mild or moderate/severe anemia completely resolved during follow-up. Group 3 = Baseline moderate/severe anemia incompletely resolved (i.e., down-modulated to mild anemia); Group 4 = Developed single episode of moderate anemia or had baseline mild anemia that persisted in two or all three follow-up intervals. Group 5 = Baseline mild anemia only or developed single episode of mild anemia in one of three follow-up intervals. Group 6 = participants with baseline moderate/severe anemia that persisted during follow-up or participants with mild baseline anemia progressed to moderate/severe during one or more follow-up intervals. Results are from linear regression models for CD4, BMI and Quality of life as outcomes. Adjusted least square means and standard error (SE) for the reference (^†^) hematologic status category are shown. For non-reference exposure categories, mean difference in outcomes from reference category and 95% confidence intervals are presented. Analyses are adjusted for age, sex, wealth and any of the following potential confounders–vitamin D, CRP, HAART naive status at enrollment, multivitamin use, alcohol use & smoking; * Additionally adjusted for baseline BMI.

**Table 4:** Time to hospitalization/death in relation to baseline ferritin status, baseline anemia, anemia severity and anemia persistence.

Hematologic Status Indicator	Number of Events/Person-Months at Risk	Unadjusted Association Hazard Ratio (95% CI)	Adjusted Association ** Hazard Ratio (95% CI)
Serum Ferritin			
High	32/2477	1.83 (1.04, 3.27)	1.75 (0.92, 3.3)
Low	11/1073	1.56 (0.73, 3.30)	1.86 (0.85, 4.1)
Normal Ferritin	18/2608	Ref	Ref
Baseline Anemia			
Present	40/2906	2.09 (1.23, 3.55)	2.03 (1.18, 3.56)
Absent	21/3388	Ref	Ref
Baseline Anemia Severity			
No Anemia	21/3388	Ref	Ref
Mild Anemia	22/1840	3.4 (1.89, 6.23)	3.9 (2.1, 7.2)
Moderate/Severe	18/1146	6.3 (3.34, 11.7)	6.7 (3.6, 12.7)
Anemia Persistence ***			
Group 1	15/2767	Ref	Ref
Group 2	6/882	1.2 (0.5, 3.2)	1.11 (0.4, 2.9)
Group 3	6/538	2.2 (0.8, 5.6)	2.1 (0.8, 5.5)
Group 4	11/862	2.27 (1.04, 4.96)	2.27 (1.01, 5.1)
Group 5	10/393	4.74 (2.1,10.6)	4.70 (2.0,10.9)
Group 6	13/753	3.0 (1.4, 6.4)	3.1 (1.4, 6.5)

** Multivariable model adjusted for: age, female sex, wealth, baseline CRP, HAART experience at enrollment, multivitamin use history, baseline vitamin D, smoking status, baseline BMI and baseline CD4; randomization to multiple micronutrient supplementation vs. placebo. *** Group 1 = adults anemia free in all intervals; Group 2 = Participants with baseline mild or moderate/severe anemia completely resolved during follow-up. Group 3 = Baseline moderate/severe anemia incompletely resolved (i.e., down-modulated to mild anemia); Group 4 = Developed single episode of moderate anemia or had baseline mild anemia that persisted in two or all three follow-up intervals. Group 5 = Baseline mild anemia only or developed single episode of mild anemia in one of 3 follow-up intervals. Group 6 = participants with baseline moderate/severe anemia that persisted during follow-up or participants with mild baseline anemia progressed to moderate/severe during one or more follow-up intervals.

## Supplementary Materials

The following are available online at http://www.mdpi.com/2072-6643/10/4/475/s1, Table S1: Descriptive statistics for repeated bi-annual assessments of anemia and respective outcomes, Table S2: Descriptive statistics for outcomes by anemia persistence at study month 18.
