# Supplementary material for: Impaired Hematologic Status in Relation to Clinical Outcomes among HIV-Infected Adults from Uganda: A Prospective Cohort Study
Source: Nutrients. 2018 Apr 12;10(4):475. doi: 10.3390/nu10040475 (PMC5946260; doi:10.3390/nu10040475)
Supplement: Supplementary file 1 [file nutrients-10-00475-s001.pdf]

Table S1: Descriptive Statistics for Repeated Bi-annual Assessments of Anemia and Respective Outcomes.

	**Month 0**	**Month 6**	**Month 12**	**Month 18**
	**Mean (SD)**	**Mean (SD)**	**Mean (SD)**	**Mean (SD)**
**Hemoglobin (mg/dL, mean, SD)**	12.3 (1.9)	12.9 (1.8)	13.2 (1.7)	13.3 (1.7)
**BMI (in kg/m^2)**	23.8 (4.4)	24.2 (4.3)	24.6 (4.2)	25.0 (4.4)
**QOL Score**	91.5 (9.9)	97.5 (9.0)	98.7 (7.9)	98.4 (8.4)
**CD4 cell count (Cells/ µL)**	148.9 (95.9)	231.7 (112.4)	262.5 (127.8)	295.8 (152.1)
**Anemia Severity**	**N (%)**	**N (%)**	**N (%)**	**N (%)**
**No Anemia**	206 (51.5)	254 (60.8)	280 (75.5)	284 (77.4)
**Mild Anemia**	113 (28.3)	89 (23.4)	64 (17.3)	60 (16.4)
**Moderate/Severe Anemia**	81 (20.3)	37 (9.7)	27 (7.3)	23 (6.3)

Table S2: Descriptive statistics for outcomes by anemia persistence at study month 18.

	Group 1 n=166	Group 2 n=58	Group 3 n=34	Group 4 n=54	Group 5 n=19	Group 6 n=38
**Analyses of Mean Responses***	**Mean (SD)**	**Mean (SD)**	**Mean (SD)**	**Mean (SD)**	**Mean (SD)**	**Mean (SD)**
CD4 cell count (Cells/ µL)	298 (154)	338 (159)	291 (117)	272 (144)	280 (127)	266 (177)
BMI (in kg/m^2)	25.2 (4.5)	24.9 (3.9)	25.2 (4.5)	24.1 (4.0)	23.4 (3.9)	25.7 (5.2)
QOL Score	98.7 (8.4)	98 (8.6)	100 (7.4)	97.5 (8.7)	99.9 (5.8)	95.7 (11.0)
**Time to event Analyses****	N=162	N=53	N=33	N=55	N=29	n=47
Hospitalized (n, %)	11 (6.8)	6 (11.3)	6 (18.2)	7 (12.7)	6 (20.7)	10 (21.3)
Died	4 (2.5)	0 (0)	0 (0)	4 (7.3)	4 (13.8)	5 (10.6)
Hospitalized or Died	15 (9.3)	6 (11.3)	6 (18.2)	11 (20.0)	10 (34.5)	13† (27.7)

Group 1 = adults anemia free in all intervals; Group 2 = Participants with baseline mild or moderate/severe anemia completely resolved during FU. Group 3 = Baseline moderate/severe anemia down incompletely resolved (i.e. down modulated to mild anemia); Group 4 = Developed single episode of moderate anemia OR baseline mild anemia persisted in two or all three follow-up intervals. Group 5 = Baseline mild anemia only or developed single episode of mild anemia in one of 3 follow-up intervals. Group 6 =participants with baseline moderate/severe anemia that persisted during follow-up or participants with mild baseline anemia progressed to moderate/Severe during one or more follow-up intervals.

*: Exposure variable defined for the entire study period and includes persons hospitalized at baseline but living as subsequent FU intervals.

*: Exposure variable defined only up to time of hospitalization or death (if no hospitalization). Surviving persons reporting hospitalization at baseline only (n=21) were removed from study base to preserve temporal sequence for exposure outcome relationship. For surviving participants reporting baseline hospitalization and another hospitalization event during follow-up, time to event was defined as time between enrollment and second hospitalization. †: Two participants were hospitalized before eventual death. Both were censored at hospitalization event.
